# NagZ modulates the virulence of *E. cloacae* by acting through the gene of unknown function, ECL_03795

**DOI:** 10.1080/21505594.2024.2367652

**Published:** 2024-06-24

**Authors:** Xianggui Yang, Jun Zeng, Dan Wang, Qin Zhou, Xuejing Yu, Zhenguo Wang, Tingting Bai, Guangxin Luan, Ying Xu

**Affiliations:** a Department of Laboratory Medicine, Clinical Medical College and the First Affiliated Hospital of Chengdu Medical College, Chengdu, Sichuan, China; b Division of Pulmonary and Critical Care Medicine, Clinical Medical College and the First Affiliated Hospital of Chengdu Medical College, Chengdu, Sichuan, China; c Department of Internal Medicine, Division of Cardiology, University of Texas Southwestern Medical Center, Dallas, TX, USA; d Department of Stomatology, Clinical Medical College and the First Affiliated Hospital of Chengdu Medical College, Chengdu, Sichuan, China

**Keywords:** *E. cloacae*, NagZ, virulence, ECL_03795, c-di-GMP

## Abstract

β-N-acetylglucosaminidase (NagZ), a cytosolic glucosaminidase, plays a pivotal role in peptidoglycan recycling. Previous research demonstrated that NagZ knockout significantly eradicated AmpC-dependent β-lactam resistance in *Enterobacter cloacae*. However, NagZ’s role in the virulence of *E. cloacae* remains unclear. Our study, incorporating data on mouse and *Galleria mellonella* larval mortality rates, inflammation markers, and histopathological examinations, revealed a substantial reduction in the virulence of *E. cloacae* following NagZ knockout. Transcriptome sequencing uncovered differential gene expression between NagZ knockout and wild-type strains, particularly in nucleotide metabolism pathways. Further investigation demonstrated that NagZ deletion led to a significant increase in cyclic diguanosine monophosphate (c-di-GMP) levels. Additionally, transcriptome sequencing and RT-qPCR confirmed significant differences in the expression of ECL_03795, a gene with an unknown function but speculated to be involved in c-di-GMP metabolism due to its EAL domain known for phosphodiesterase activity. Interestingly, in ECL_03795 knockout strains, a notable reduction in the virulence was observed, and virulence was rescued upon complementation with ECL_03795. Consequently, our study suggests that NagZ’s function on virulence is partially mediated through the ECL_03795→c-di-GMP pathway, providing insight into the development of novel therapies and strongly supporting the interest in creating highly efficient NagZ inhibitors.

## Introduction

The *Enterobacter cloacae* complex (ECC) is a prevalent hospital-acquired pathogen capable of causing various infections, such as pneumonia, urinary tract infections, and septicaemia [1]. ECC comprises seven species: *Enterobacter cloacae*, *Enterobacter hormaechei*, *Enterobacter kobei*, *Enterobacter asburiae*, *Enterobacter nimipressuralis*, *Enterobacter ludwigii* and *Enterobacter mori*. Additionally, recently identified species including *Enterobacter chengduensis*, *Enterobacter* roggenkampii, and *Enterobacter bugandensis*, cluster with ECC species [2–4]. Among ECC, *E. cloacae* and *E. hormaechei* are the most commonly isolated species in clinical infections, particularly among immunocompromised patients and those hospitalized in intensive care units [5]. Studies indicate that *E. cloacae* is responsible for 5% of hospital-acquired sepsis, 5% of hospital-acquired pneumonia, 4% of hospital-acquired urinary tract infections, and 10% of postoperative peritonitis [6]. Therefore, *E. cloacae* has emerged as a significant concern for global healthcare institutions as a key pathogen. Furthermore, *E. cloacae* is well-known for its tendency to develop antibiotic resistance, driven by mechanisms such as the intrinsic cephalosporinase AmpC and transferable β-lactamases [7]. These mechanisms pose significant challenges in effectively treating *E. cloacae* infections. In this regard, apart from recently developed formulations and combinations involving β-lactamase inhibitors, innovative alternatives, including the emerging concept of antivirulence therapies, are gaining importance as promising approaches to combat bacterial infections [8].

Antivirulence therapies, aimed at diminishing the virulence of bacteria, hold the potential to enhance susceptibility to immune clearance and improve clinical outcomes for patients [9–11]. Exploring the intricate interplay between resistance and virulence, a subject of enduring interest, may serve as a foundation for these therapeutic avenues [12,13]. For instance, in the case of *Pseudomonas aeruginosa* characterized by hyperproduction of AmpC cephalosporinase, the inhibition of peptidoglycan recycling has demonstrated a significant reduction in both resistance and virulence [14].

NagZ, a cytosolic glucosaminidase, plays a pivotal role in peptidoglycan recycling. Its primary function involves hydrolysing N-acetylglucosaminy-1,6-anhydromuropeptides (peptidoglycan monomers) into N-acetylglucosaminyl (GlcNAc) and 1,6-anhydromuropeptides (anhMurNAc) [15]. Current research on NagZ primarily focuses on its role in regulating bacterial resistance. For instance, inhibiting NagZ has been demonstrated to decrease bacterial resistance to β-lactam antibiotics in various bacteria, including *Pseudomonas aeruginosa*, *Stenotrophomonas maltophilia*, and *Yersinia enterocolitica* [16–19]. Our previous research has substantiated NagZ’s critical role in regulating the expression of chromosomal AmpC β-lactamase and subsequent resistance in *E. cloacae* [20]. Additionally, limited studies have also reported NagZ’s involvement in bacterial biofilm synthesis and virulence regulation, such as NagZ triggers *Neisseria gonorrhoeae* biofilm disassembly [21] and knockout of NagZ disrupts the peptidoglycan recycling pathway, leading to reduced virulence in *Pseudomonas aeruginosa* [14,22]. Despite these significant research findings, the mechanisms underlying NagZ’s involvement in virulence regulation, particularly in *E. cloacae*, remain unclear.

In this study, we initially observed a significant reduction in the virulence of *E. cloacae* following the knockout of NagZ. Furthermore, our findings revealed that NagZ regulates virulence in *E. cloacae* through the ECL_03795 → c-di-GMP pathway. These results suggest that NagZ could be a promising target for the development of antivirulence and antibacterial therapies, offering potential new avenues for treating *E. cloacae* infections.

## Materials and methods

### Bacterial strains and cell lines

To investigate the factors effecting the virulence of *E. cloacae*, three clinically isolated strains confirmed to be pathogenic were included in this study. These strains were obtained from blood, urine, and wound secretions, and were designated as WT1, WT2, and WT3, respectively. Detailed information about these strains is listed in Table 1. NagZ knockout mutants of WT1, WT2, and WT3 were generated using the suicide vector homologous recombination technique [23], and they were denoted as Δ*nagZ*1, Δ*nagZ*2, and Δ*nagZ*3, respectively. These strains were routinely cultured in Luria-Bertani broth (BD Difco, Detroit, MI, USA) at a temperature of 37°C. The RAW264.7 macrophage-like cell line was obtained from the Shanghai Institute of Biochemistry and Cell Biology (Shanghai, China) and cultured in RPMI medium supplemented with 10% (v/v) foetal bovine serum (Gibco, Grand Island, New York).

**Table 1.** Isolates and clinical data.

strains	patients’ information			sequence type	sample	infection symptoms before treatment	using antibiotics	after using antibiotics	
	age	gender	diagnose					same pathogen detected again	symptoms of infection
WT1	72	male	sepsis	ST 696	blood	yes	yes	no	improved
WT2	68	female	cystitis	ST 422	urine	yes	yes	no	improved
WT3	53	male	traumatic infection	ST 414	wound secretion	yes	yes	no	improved

### MLST analysis

A multilocus sequence typing (MLST) methodology was applied to classify *E. cloacae* isolates into distinct clonal groups. This approach involved the analysis of seven conserved housekeeping genes (*dnaA, fusA, gyrB, leuS, pyrG, rplB*, and *rpoB*), as described by Miyoshi-Akiyama and colleagues [24]. By examining the specific combination of alleles at these seven loci, the sequence type (ST) for each isolate was determined, as defined on the MLST website (http://pubmlst.org/ecloacae/).

### Mice

BALB/c mice (6–8 weeks), purchased from Dashuo Animal Technology (Chengdu, China), were utilized for all animal experiments conducted in this study. The mice were housed in a specific pathogen-free environment, following a 12-hour light/dark cycle, at a controlled temperature (22 ± 2 °C), and maintained at a relative humidity of 50 ± 5%. To establish a reliable mouse infection model for evaluating the virulence of *E. cloacae*, we first determined the median lethal dose 50 (LD50) of three strains of *E. cloacae* (WT1, WT2, WT3). In brief, these strains were cultured in LB medium, and once they reach the exponential growth phase, the bacteria are harvested by centrifugation. Subsequently, bacterial suspensions with a density of 4.0 McFarland units (MCF) were prepared using the Maxwell turbidity method. These suspensions were then subjected to serial 10-fold dilutions, resulting in six dilutions. Finally, mice aged 6 to 8 weeks and weighing 25 to 30 g were intraperitoneally injected with various concentrations of the suspensions at a dose of 0.1 mL/10 g (body weight). The mortality, behavioural changes, and weight fluctuations of the mice were observed for the subsequent 7 days. Utilizing the Karber method [25], LD50 values for the three bacterial strains were determined to be 0.78 × 10^8^ colony-forming units (CFUs) for WT1 strain, 0.83 × 10^8^ CFUs for WT2, and 0.89 × 10^8^ CFUs for WT3. Consequently, approximately 10^8^ CFUs was chosen for assessing bacterial virulence in mouse survival experiments. To ensure the survival and welfare of mice, approximately 10^6^ CFUs of the bacterial strain were utilized for experiments involving histopathological examination, as well as the measurement of IL-6 and TNF-α expression levels. Blood, lung, liver, and spleen samples were harvested at 6 hours following infection. To maintain data integrity and minimize any potential distress to the mice, all mice experiments underwent repeated iterations. If the results from both rounds of experimentation were consistent, the experiments were deemed conclusive. In instances where discrepancies emerged between the outcomes of the two sets, a third round of experiments was carried out.

### Galleria mellonella larvae survival experiment

The virulence of the strains was assessed using *Galleria mellonella* larvae, following established protocols [26]. Cultures of the respective strains in exponential growth phase were harvested, washed, and resuspended in PBS. Approximately 150 CFUs of the strains were injected into individual research-grade *Galleria mellonella* larvae through the hindmost left proleg using Hamilton syringes (Hamilton Industrial Technology CO., Ltd. Tianjin, China). Each strain was injected into 10–15 larvae to ensure experimental reliability. The survival of infected *Galleria mellonella* larvae was monitored every 6 hours for 120 hours. As controls, 10–15 larvae were inoculated with 10 μL of PBS. The procedure was repeated three times.

### RNA extraction and RT-qPCR assays

Total RNA extraction from *E. cloacae* isolates was carried out using the RNA kit (Sangon Biotech Co., Ltd. Shanghai, China) following the manufacturer’s protocol. The concentration of the extracted total RNA was determined using a NanoDropTM8000 spectrophotometer (Thermo Fisher Scientific, Waltham, Mass, USA). The total RNA was stored at −70°C for subsequent gene expression analysis. cDNA synthesis was performed using the FastKing gDNA Dispelling RT SuperMix kit (Tiangen Biotech Co., Ltd. Beijing, China) with 500ng of total RNA as the template. Real-time fluorescence quantitative PCR (qPCR) was conducted using the SuperReal PreMix Color (SYBR Green) kit (Tiangen Biotech Co., Ltd. Beijing, China) following the manufacturer’s instructions. The 16S gene was used as the internal control for qPCR assays. The final primer concentration in each reaction was 0.25 μM. The sequences of the primers used are listed in Table 2. The experiment was conducted three times.

**Table 2.** Primers information.

Primers	Primer sequences		Product size(bp)
ECL_03795-qPCR Forward	5’GGGGTTATTGCCGATCTGTC 3’	136	
ECL_03795-qPCR Reverse	5’GTTGTTGTCGAGCGCCTTTT 3’		
ECL_02048-qPCR Forward	5’CCTGAACTGGCGAGAATGGC 3’	143	
ECL_02048-qPCR Reverse	5’CCTGAACTGGCGAGAATGGC 3’		
*nagZ*-CDS Forward	5’ATGTTGGATGTCGAAGGGTTTG 3’	1014	
*nagZ*-CDS Reverse	5’TTAAAGGGCTGCTTTATGTGCC 3’		
ECL_03795-CDS Forward	5’ATGAACACTATCGCTTTCCTG 3’	2241	
ECL_03795-CDS Reverse	5’TCAGGCGTCCGCTACGGACG 3’		
16S-qPCR Forward	5’TCCTACGGGAGGCAGCAGT 3’	467	
16S-qPCR Reverse	5’GGACTACCAGGGTATCTAATCCTGTT 3’		

qPCR: Real-time fluorescence quantitative polymerase chain reaction, CDS: coding DNA sequence.

### Protein extraction and Western blot analysis

*E. cloacae* strains were cultured overnight in LB medium at 37°C and 250rpm and the overnight bacterial culture was then sub-cultured in fresh LB medium at a dilution of 1:100. Once the OD600 absorbance reached 0.8, the bacterial cells were harvested and suspended in 1 ml of protein lysis buffer (Sangon Biotech Co., Ltd. Shanghai, China). The samples were sonicated to facilitate cell lysis and then centrifuged at 10,000 g for 10 minutes to collect the supernatant. The protein concentration was determined using a protein quantification kit (Beyotime Biotechnology, Shanghai, China). For Western blot analysis, 30 μg of protein was used. The Western blot analysis was performed following the standard methodology as previously reported [27]. The following antibodies were used: rabbit anti-NagZ (prepared in-house), mouse anti-DnaK (Abcam, Cambridge, MA, USA), goat anti-mouse IgG-HRP, and goat anti-rabbit IgG-HRP (Santa Cruz Biotechnology, Inc., Santa Cruz, CA, USA). Images were captured using a SPOT-CCD camera, and DnaK was used as the internal control. The experiment was repeated three times.

### Construction of nagZ and ECL_03795 knockout *E.* *cloacae* models

The construction of *nagZ* and ECL_03795 knockout *E. cloacae* models followed a previously described method involving suicide vector homologous recombination [23]. Initially, two DNA fragments corresponding to the homologous arms of the *nagZ* and ECL_03795 genes were individually amplified through PCR. Subsequently, these distinct homologous arms were fused via fusion PCR, generating separate fusion DNA fragments for each gene. These fusion DNA fragments were then individually cloned into the suicide plasmid pLP12 and underwent verification through sequencing and PCR analysis. The resulting recombinant plasmids carrying the respective fusion DNA fragments were separately introduced into *Escherichia coli* β2163. The *nagZ* and ECL_03795 knockout strains were obtained by co-culturing *Escherichia coli* β2163 with the recombinant plasmids and wild-type *E. cloacae*. All necessary reagents and strains for constructing the *nagZ* and ECL_03795 knockout *E. cloacae* models were sourced from Nuojing Biological Company (Guangzhou, China).

### Preparation of EC models for NagZ and ECL_03795 complementation

The coding DNA sequences (CDS) of *nagZ* and ECL_03795 were amplified via PCR, subsequently cloned into the pBAD33cm-rp4 plasmid, and confirmed by sequencing. The resulting recombinant plasmids (pBAD33-*nagZ* and pBAD33-ECL_03795) were then introduced into competent *Escherichia coli* β2163 cells. Subsequently, the recombinant plasmid from *Escherichia coli β2163* was transferred into *E. cloacae* using a conjugation assay. Induction of *nagZ* and ECL_03795 gene expression was achieved by supplementation with 0.05% L-Arabinose (Sangon Biotech Co. Ltd, Shanghai, China). All plasmids and strains used in this study were obtained from Nuojing Biological Company. The primer sequences for obtaining the CDS of *nagZ* are listed in Table 2.

### Transcriptomic analysis

#### RNA sequencing and data analysis

Gene expression analysis utilized Illumina RNA sequencing (RNA-Seq) technology. Each sample underwent RNA-Seq with three biological replicates. Libraries were generated using the Illumina TruSeq stranded mRNA sample prep kit, and sequencing was performed on the Illumina HiSeq 2500 platform (Illumina, USA) using a paired-end protocol with 100-nucleotide read lengths. Approximately 20 million reads were obtained for each sample. Trimming excluded adaptor sequences and reads below 50 bp. Trimmed reads were aligned to the reference genome of *E. cloacae* standard strain ACTT 13,047. Unique sequence reads were filtered based on specific criteria, including a maximum hit count of 1 per read, a minimum length fraction of 0.9, a minimum similarity fraction of 0.8, and a maximum of 2 mismatches. To avoid issues caused by zero values, a constant value of 1 was added to the raw transcript count. Differential expression analysis was conducted using the Deseq2 R package [28]. Raw counts were normalized based on the library size of each sample. A negative binomial test was performed to identify genes with differential expression. Transcripts were considered differentially expressed in pairwise comparisons if their absolute fold change exceeded 2 and the associated adjusted *p* value was below 0.05. Prior to performing principal component analysis (PCA), the normalized transcripts were log2-transformed with the addition of a pseudocount of 1.

#### Functional enrichment of E. cloacae transcriptome

To analyse the functional enrichment of the *E. cloacae* transcriptome in terms of biological processes, we utilized WebGestalt [29,30]. The transcriptomic data of *E. cloacae*, including gene symbols and fold changes, were used as input. *E. cloacae* was chosen as the reference organism for the analysis. Gene Set Enrichment Analysis (GSEA) was performed on the uploaded genes, using either the Kyoto Encyclopedia of Genes and Genomes (KEGG) pathway or Gene Ontology (GO) pathway as functional databases.

### Biofilm formation assays

Biofilm formation was evaluated by employing crystal violet staining [31]. In brief, *E. cloacae* strains were cultured overnight and subsequently diluted in fresh LB medium to an OD600 of 0.05. The diluted cell suspension was then divided into either sterile 96-well polystyrene microtiter plates (200 μl per well) or tubes (5 ml per tube). Following 24 hours of incubation at room temperature without shaking, the culture supernatant was discarded, and the wells or tubes were rinsed twice with sterile water. The attached cells were stained with a 0.1% (w/v) crystal violet solution for 15 minutes, followed by two washes with distilled water to remove unbound dye. For the 96-well plates, the dye bound to the bacteria was solubilized using 200 μl of 95% ethanol, and the absorbance was measured at 590 nm using a microplate reader (Biotek, Vermont, USA). The biofilm levels were normalized based on the bacterial growth rate for each sample. The experiment was repeated three times.

### Cytokine analysis

In the cellular stimulation experiment, RAW 264.7 cells were plated at a density of 10^5^ cells per well in a 24-well microtiter plate. Subsequently, the cells were infected with wild-type *E. cloacae* (WT), *nagZ* knockout *E. cloacae* (Δ*nagZ*), Δ*nagZ* complemented with NagZ (ΔnagZ+NagZ), ECL_03795 knockout *E. cloacae* (Δe03795), and Δe03795 complemented with ECL_03795 (Δe03795+ECL_03795). After 6 hours of incubation, the supernatant was collected, and the levels of IL-6 and TNF-α were quantified using an enzyme-linked immunosorbent assay (ELISA) kit from Sangon Biotech Co. Ltd (Shanghai, China), according to the manufacturer’s instructions. The experiment was repeated three times. In the mouse infection experiment, BALB/c mice were inoculated with the aforementioned strains at a dosage of 10^6^ CFUs. After 6 hours, blood samples were collected from the mice, and the levels of IL-6 and TNF-α in the mouse serum were measured using ELISA kits from Sangon Biotech Co. Ltd. To ensure data integrity and minimize mouse distress, experiments underwent iterative validation; consistent results deemed them conclusive, while discrepancies prompted further rounds of experimentation.

### Levels of c-di-GMP analysis

The target strains were cultured overnight in LB medium at 37°C and 250 rpm and were then sub-cultured in fresh LB medium at a dilution of 1:100. When the OD600 absorbance reached 1.3, the supernatant was collected by centrifugation. The levels of c-di-GMP were determined using an ELISA kit from Mlbio Biotech Co. Ltd (Shanghai, China). The levels of c-di-GMP were normalized to the number of bacterial cells for each sample. The procedure was repeated three times.

### Statistical analysis

The data were presented as mean ± standard deviation. Statistical analysis comparing two groups was performed using GraphPad Prism 5 with a two-tailed t-test. Significance levels were denoted as follows: *p < 0.05, **p < 0.01, and ***p < 0.001, representing statistically significant, highly significant, and extremely high statistical differences, respectively.

## Results

### Isolates selection and construction of nagZ knockout

*E. cloacae* (EC), a common opportunistic pathogen frequently found in the human intestinal tract, was selected for studying the factors regulating its virulence. Here, three EC strains isolated from blood (WT1), urine (WT2), and wound secretions (WT3), respectively, which were clinically confirmed as pathogenic bacteria, were selected. Detailed clinical information about these strains is provided in Table 1. To investigate the role of NagZ in EC virulence, a suicide vector homologous recombination technique was employed. Following previously reported [23], *nagZ* knockout strains were individually constructed for WT1, WT2, and WT3, designated as Δ*nagZ*1, Δ*nagZ*2, and Δ*nagZ*3, respectively. As illustrates in Supplementary Figure S1, western blot assay has confirmed the mutant strains of Δ*nagZ*1, Δ*nagZ*2, and Δ*nagZ*3 has been successful constructed.

### Significant decrease in virulence of nagZ knockout EC

**Figure 1.**
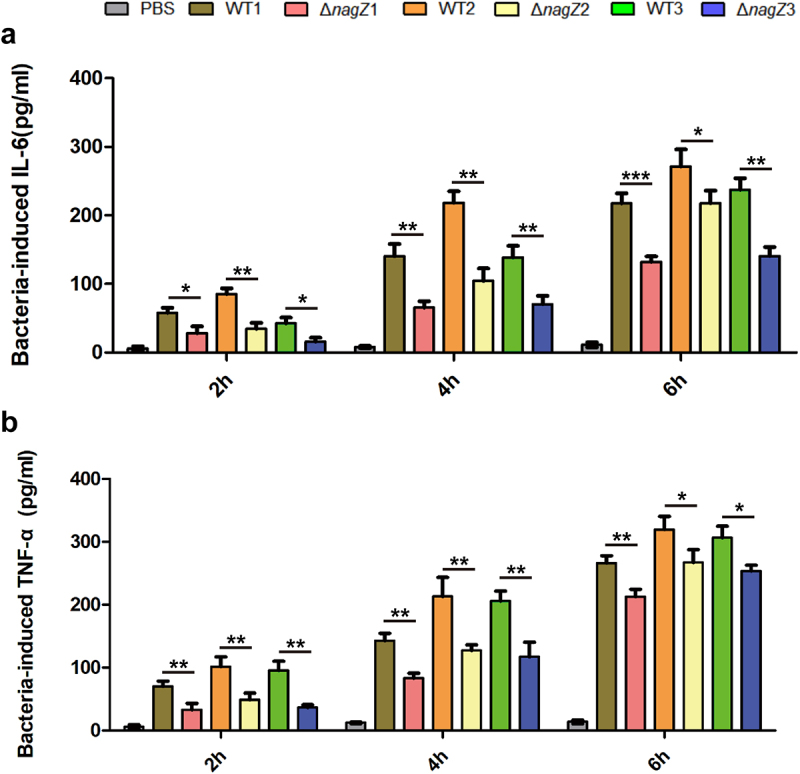
Depletion of NagZ attenuates the inflammatory response of murine macrophages RAW264.7 to *E.* *cloacae*. (a) Variations in IL-6 expression at different time points following *E.* *cloacae* infection in murine macrophages RAW264.7. (b) Changes in TNF-α expression at different time points after *E.* *cloacae* infection in murine macrophages RAW264.7. * (p < 0.05) indicates a statistically significant, **(p < 0.01) denotes a high level of statistical significance and ***(p < 0.001) signifies extremely high statistical difference (p < 0.001). WT: wild type *E.* *cloacae*, Δ*nagZ*: *nagZ* knockout *E.* *cloacae*.

Firstly, we investigated NagZ’s role in inflammation regulation *in vitro*. Mouse macrophages (RAW 264.7) were co-cultured with various strains, including WT1, WT2, WT3, Δ*nagZ*1, Δ*nagZ*2, and Δ*nagZ3*. Subsequently, we assessed the expression levels of the proinflammatory cytokines IL-6 and TNF-α in the cell culture supernatant at 2, 4, and 6 hours post-infection. Our findings demonstrated a notable decrease in the ability of Δ*nagZ* strains to stimulate IL-6 and TNF-α expression when compared to WT strains, especially during the initial 4-hour timeframe, leading to an approximately 50% reduction in expression levels (Figure 1).

**Figure 2.**
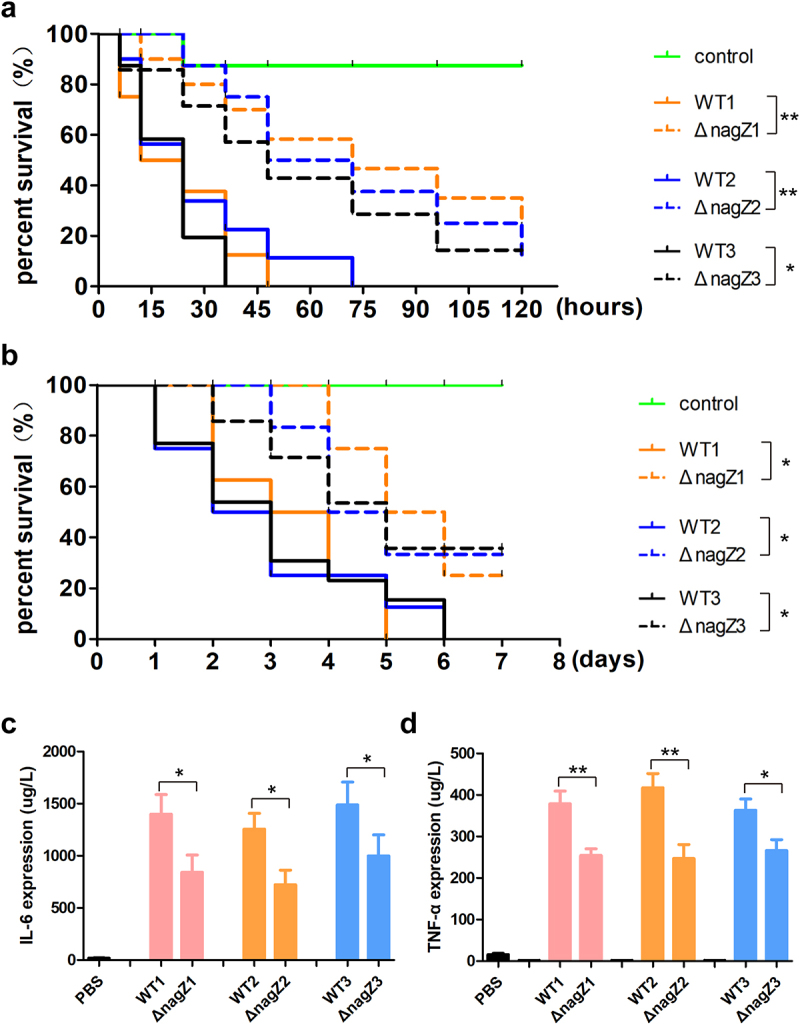
NagZ knockout diminishes *E.* *cloacae* virulence. (a, b) the survival rates of *Galleria mellonella* larvae (a) and mice (b) infected with *nagZ*-knockout *E.* *cloacae* are significantly improved compared to those infected with wild-type *E.* *cloacae.* (c, d) the expressions of IL-6 and TNF-α are downregulated in the serum of mice infected by *nagZ*-knockout *E.* *cloacae*, in contrast to wild-type *E.* *cloacae*. *p < 0.05 and ** p < 0.01 indicate statistically significant and statistically highly significant, respectively.

Subsequently, to evaluate the function of NagZ on EC virulence, we employed *Galleria mellonella* larvae and mice as model organisms. Examination of mortality rates demonstrated a substantial decrease in both *Galleria mellonella* larvae and mice infected with Δ*nagZ* strains in comparison to those infected with WT strains (Figure 2(a,b)). This underscores the crucial role of NagZ as a regulatory factor in EC virulence.

Finally, to investigate the effects of NagZ on inflammation *in vivo*, we established a systemic infection model in mice utilizing strains WT1, WT2, WT3, Δ*nagZ*1, Δ*nagZ*2, and Δ*nagZ*3. Following a six-hour post-infection period, the IL-6 and TNF-α levels in mouse serum were quantified. The findings revealed a noteworthy reduction in the Δ*nagZ*-infected group when contrasted with the WT-infected group (Figure 2(c,d)). Furthermore, we conducted haematoxylin-eosin staining to observe inflammation in organs with abundant blood supply, such as the lungs, liver, and spleen. It became apparent that mice infected with WT strains exhibited more pronounced inflammation in these organs compared to those infected with Δ*nagZ* strains (Supplementary Figure S2). These experiments, comprising both *in vitro* and *in vivo*, provide compelling evidence supporting NagZ as a major regulatory factor in EC virulence.

### Complementation of NagZ partially rescues the virulence of ΔnagZ strains

**Figure 3.**
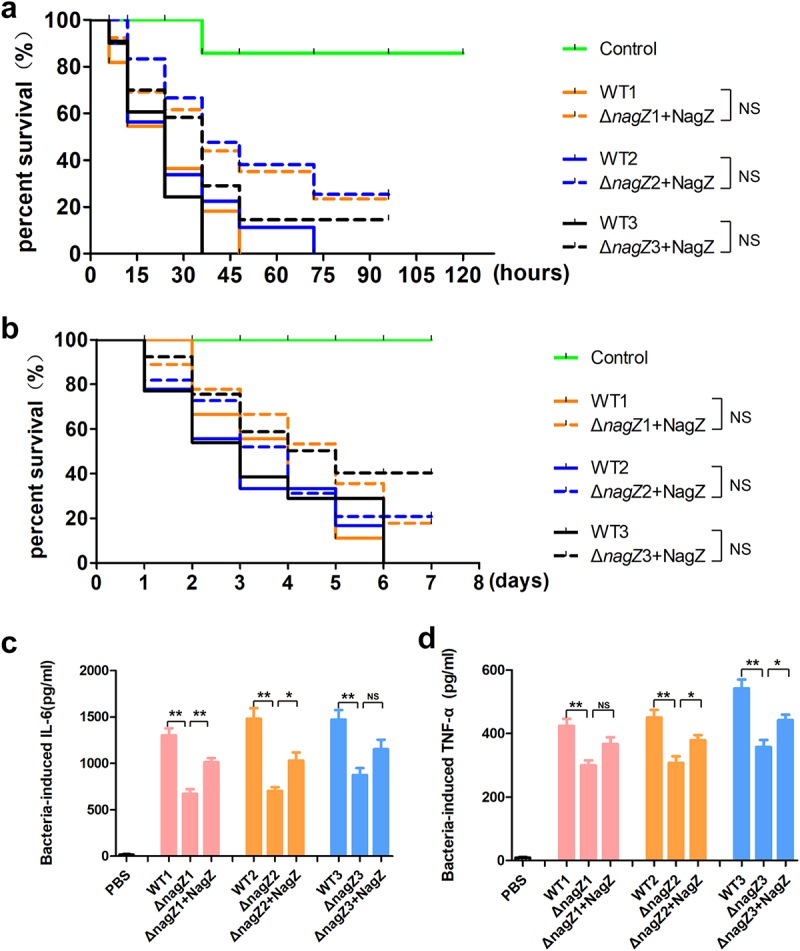
NagZ complementation partially rescues *E.* *cloacae* virulence in *nagZ* knockout models. (a,b) NagZ complementation decreases the survival rate of *Galleria mellonella* larvae (A) and mice (B). (c,d) the expression levels of IL-6 (C) and TNF-α (D) are upregulated in the serum of mice infected by *E.* *cloacae* complemented with NagZ. Δ*nagZ*+NagZ: Δ*nagZ* strains with NagZ complementation. * (p < 0.05) indicates a statistically significant, and **(p < 0.01) denotes a high level of statistical significance. NS: none statistical significance.

To gain deeper insights into the role of NagZ in regulating EC virulence, we introduced NagZ into the Δ*nagZ* strains, creating NagZ-complemented strains (Δ*nagZ*+NagZ). Subsequently, both *Galleria mellonella* larvae and mice were separately infected with WT strains and Δ*nagZ*+NagZ strains. Analysis of survival curves revealed a significant increase in mortality rates for both mice and *Galleria mellonella* larvae infected with Δ*nagZ*+NagZ strains (Figure 3(a,b)). Although the mortality rates of *Galleria mellonella* larvae and mice in the Δ*nagZ*+NagZ-infected group were slightly lower than those in the WT-infected group, there was no statistically significant difference between the two groups (Figure 3(a,b)). Moreover, in the mouse bloodstream infection model, the Δ*nagZ*+NagZ strains exhibited a stronger capacity to stimulate the expression of proinflammatory cytokines IL-6 and TNF-α compared to the Δ*nagZ* strains, although it still did not reach the level observed with the WT strains (Figure 3(c,d)). These findings suggest that NagZ complementation partially enhances the virulence of Δ*nagZ* strains.

### Transcriptome sequencing analysis unveiled a potential role of NagZ in ribonucleotide metabolism, particularly in purine ribonucleotide metabolism

**Figure 4.**
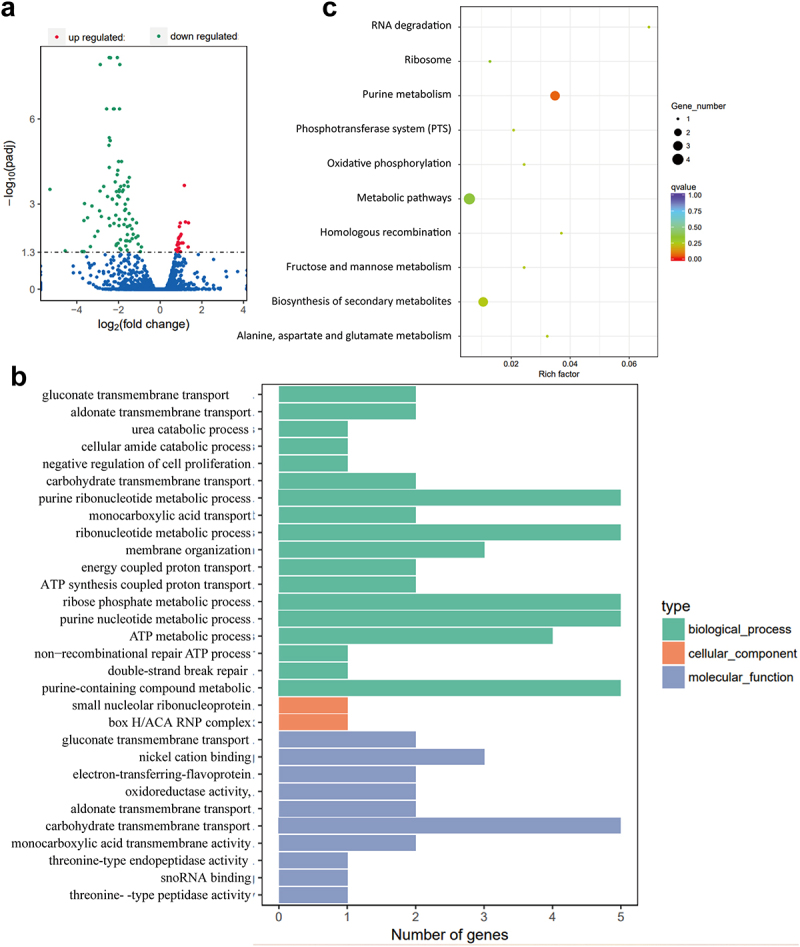
Transcriptome sequencing reveals NagZ’s involvement in nucleotide metabolism, specifically purine nucleotide metabolism. (a) NagZ knockout upregulates 24 genes and downregulates 86 genes in *E.* *cloacae*. (b) Gene ontology (GO) enrichment analysis assesses the impact of *nagZ* knockout on metabolic pathways in *E.* *cloacae*. (c) Kyoto encyclopedia of genes and genomes (KEGG) enrichment analysis examines the effect of *nagZ* knockout on metabolic pathways in *E.* *cloacae*.

To explore the mechanisms influencing *E. cloacae*‘s virulence under NagZ regulation, we conducted transcriptome sequencing and compared the genes expression profile of WT strains and Δ*nagZ* strains. This analysis revealed 110 genes with statistically significant differences (*p* < 0.05) between WT and Δ*nagZ* strains, comprising 24 upregulated genes and 86 downregulated genes (Figure 4a). Subsequently, we conducted GO enrichment analysis on the differentially expressed genes (DEGs). The results, illustrated in Figure 4b and detailed in Table S1 (results of GO enrichment analyses of the DEGs), revealed a notable enrichment in the ribonucleotide metabolism pathway, particularly in purine ribonucleotide metabolism. Furthermore, KEGG enrichment analysis also highlighted a significant enrichment in the purine metabolism pathway among these DEGs, as depicted in Figure 4c and described in Table S2 (results of KEGG enrichment analyses of the DEGs). These findings suggest that NagZ likely plays a pivotal role in ribonucleotide metabolism, with a particular focus on purine ribonucleotide metabolism.

### NagZ is involved in the synthesis of c-di-GMP and the formation of biofilm

**Figure 5.**
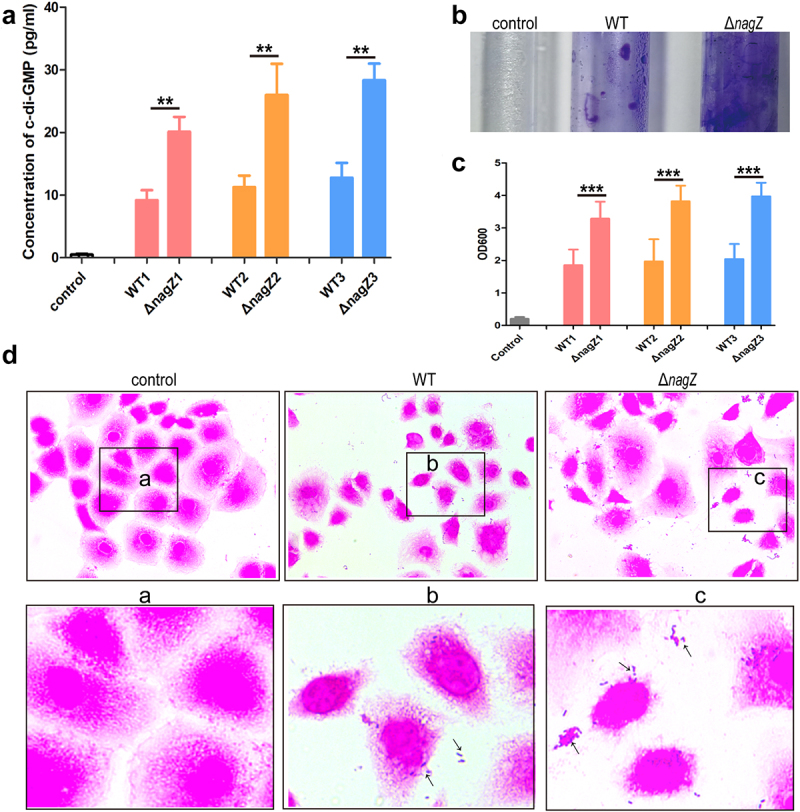
NagZ’s role in *E.* *cloacae* biofilm synthesis. (a) *nagZ* knockout enhances the synthesis of the second messenger c-di-GMP. (b) *nagZ* knockout increases *E.* *cloaca*e biofilm synthesis, as observed through crystal violet staining. (c) Quantitative analysis of NagZ’s role in biofilm synthesis conducted via crystal violet staining experiments. (d) Detection of NagZ’s impact on *E.* *cloacae* adhesion to organic material (HEK-293T cells). * (p < 0.05) indicates a statistically significant, **(p < 0.01) denotes a high level of statistical significance and ***(p < 0.001) signifies extremely high statistical difference (p < 0.001).

Transcriptome sequencing analysis uncovered NagZ’s potential involvement in purine ribonucleotide metabolism in EC. Previous studies have established c-di-GMP, a versatile second messenger derived from guanosine triphosphate (GTP), as a key player in various biological processes, including virulence and invasion [32]. Therefore, we speculate that NagZ may contribute to the synthesis of c-di-GMP. To confirm our hypothesis, we assessed c-di-GMP levels in the culture supernatant of both the WT and Δ*nagZ* strains. Strikingly, we detected a significant increase in c-di-GMP synthesis and release in the Δ*nagZ* strains compared to the WT strains (Figure 5a). Research has shown that low c-di-GMP levels enhance bacterial motility and virulence factor secretion, while high concentrations promote bacterial sessility and facilitate biofilm formation [33,34]. Consequently, we further explored NagZ’s impact on biofilm formation using crystal violet staining. The result revealed that the Δ*nagZ* strains exhibited a higher propensity for biofilm formation compared to the WT strains (Figure 5(b,c)). Studies have demonstrated that enhanced biofilm formation indicates stronger bacterial adhesion [33]. Therefore, we evaluated the adhesion capacities of Δ*nagZ* strains and WT strains to HEK-293T cells. The finding demonstrated that the NagZ deletion significantly enhanced *E. cloacae* ‘s adhesion to HEK-293T cells (Figure 5d). In summary, these data provide compelling evidence that NagZ modulates *E. cloacae* biofilm formation through c-di-GMP, thereby influencing its adhesion capabilities.

### NagZ plays a role in regulating the gene ECL_03795, a gene with an unknown function but speculated to be associated with c-di-GMP metabolism

**Figure 6.**
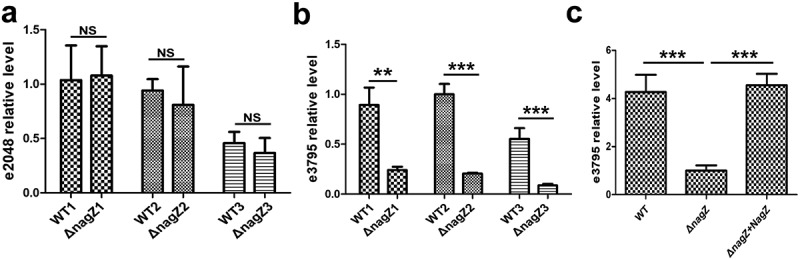
Illustrating NagZ’s role in the expression levels of phosphodiesterases EC_02048 and EC_03795. (a) RT-qPCR results indicate that NagZ does not significantly affect the expression of EC_02048. (b) After NagZ knockout, there is a substantial downregulation in the expression level of phosphodiesterases EC_03795. (c) The expression level of phosphodiesterases EC_03795 is rescued upon complementation of NagZ in *nagZ*-knockout *E.* *cloacae*. e2048: ECL_03795 gene, e3795: ECL_03795 gene. * (p < 0.05) indicates a statistically significant, and **(p < 0.01) denotes a high level of statistical significance and ***(p < 0.001) signifies extremely high statistical difference (p < 0.001).

In bacteria, c-di-GMP levels are typically regulated by two enzyme types: diguanylate cyclases (DGCs), responsible for c-di-GMP synthesis, and phosphodiesterases, primarily responsible for c-di-GMP degradation. In the transcriptome sequencing analysis, two genes with unknown functions, namely ECL_02048 and ECL_03795, were found to be differentially expressed in both the WT and Δ*nagZ* strains (Supplementary Table S3). While their functions remain uncharacterized, there is speculation that these genes could be linked to c-di-GMP metabolism due to their featuring an EAL domain known for its phosphodiesterase activity. RT-qPCR results, as shown in Figure 6, reveal a significant decrease in ECL_03795 expression following *nagZ* knockout (Figure 6b), while ECL_02048 expression remains unchanged (Figure 6a). What’s particularly intriguing is that NagZ complementation rescues the downregulated expression of ECL_03795 resulting from *nagZ* deletion in the Δ*nagZ* strain (Figure 6c). Hence, it is plausible that NagZ is implicated in the regulation of c-di-GMP through the putative phosphodiesterase ECL_03795.

### Knockout of the ECL_03795 rescues the reduced virulence caused by *nagZ* knockout

**Figure 7.**
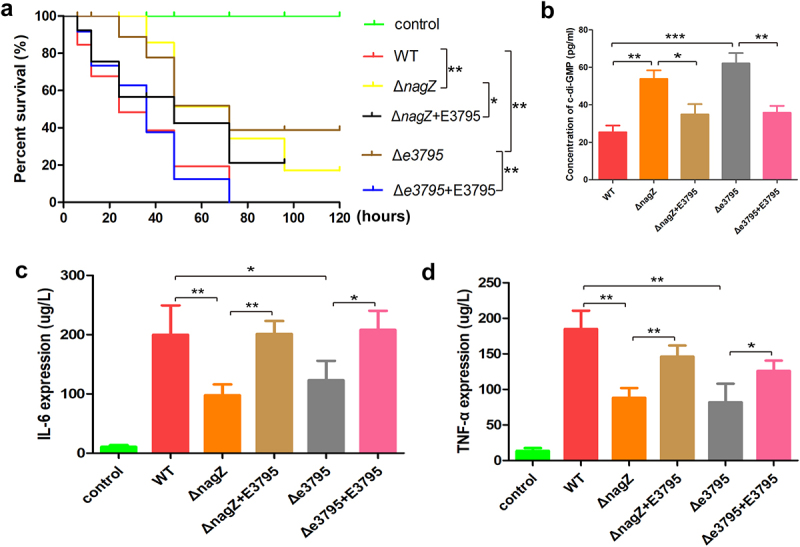
Examining the roles of NagZ and EC_03795 in *E.* *cloacae* virulence. (a) The survival rate of *Galleria mellonella* larvae assay assesses the impact of NagZ and EC_03795 on *E.* *cloacae’*s virulence. (b) Illustrates the functions of NagZ and EC_03795 in the expression of the second messenger c-di-GMP in *E.* *cloacae*. (c, d) evaluating the roles of NagZ and EC_03795 in the expression of the inflammatory factors IL-6 (C) and TNF-α (D) in a mouse infection model. Δ*nagZ*+E3795: Δ*nagZ* strain complemented with ECL_03795, Δe3795: WT1 strain with knockout of the ECL_03795 gene, Δe3795+E3795: Δe3795 strain complemented with ECL_03795. * (p < 0.05) indicates a statistically significant, **(p < 0.01) denotes a high level of statistical significance and ***(p < 0.001) signifies extremely high statistical difference (p < 0.001).

As mentioned above, ECL_03795 encodes a putative phosphodiesterase. Consequently, we hypothesized that NagZ’s influence on the virulence of *E. cloacae* might occur via the ECL_03795 → c-di-GMP pathway. To investigate this, we generated an ECL_03795 knockout strain in the WT background (Δe3795) and complemented ECL_03795 into Δ*nagZ* (Δ*nagZ*+E3795) and Δe3795 (Δe3795+E3795), respectively. *Galleria mellonella* larvae survival experiments demonstrated a significant reduction in virulence in the Δe3795 strain compared to the WT strain. Conversely, complementing ECL_03795 in the Δe3795 strain markedly enhanced *E. cloacae*‘s virulence. Intriguingly, complementing ECL_03795 into the Δ*nagZ* strain also resulted in increased virulence (Figure 7a).

Furthermore, we established a mouse bloodstream infection model using WT, Δ*nagZ*, Δ*nagZ*+E3795, e3795, and Δe3795+E3795 strains. After 6 hours of infection, we measured the expression levels of the proinflammatory cytokines IL-6 and TNF-α. Consistent with the findings from the *Galleria mellonella* larvae survival experiments, knocking out ECL_03795 significantly reduced the ability of the WT strain to induce IL-6 and TNF-α expression in mice. In contrast, complementing ECL_03795 in Δe3795 strain rescued the expression of IL-6 and TNF-α in mice (Figure 7(c,d)). All those data confirmed ECL_03795’s involvement in regulating *E. cloacae*‘s virulence.

To further identify the mechanism through which ECL_03795 regulates *E. cloacae*‘s virulence, we investigated ECL_03795’s role in c-di-GMP regulation. Similar to *nagZ* mutant, ECL_03795 knockout increased c-di-GMP synthesis in *E. cloacae*, while complementing ECL_03795 inhibited c-di-GMP synthesis (Figure 7b). These results confirm that NagZ’s regulation for virulence of *E. cloacae* is mediated through the ECL_03795 → c-di-GMP pathway.

## Discussion

As widely recognized, the deletion of NagZ is a well-established method for inhibiting the recycling of N-acetylglucosamine (NAG) and N-acetylmuramyc acid (NAM), which currently stands as the primary approach to disrupt peptidoglycan recycling [35]. In the case of *Pseudomonas aeruginosa*, previous studies have reported a significant reduction in β-lactam resistance when peptidoglycan recycling is impeded [13,36]. Our earlier research findings also pointed to a noticeable decrease in β-lactam resistance in *E. cloacae* following NagZ deletion [20]. Nevertheless, the impact of NagZ on the virulence of *E. cloacae* remains unclear. In this study, we first observed a significant decrease in the virulence of *E. cloacae* due to the absence of NagZ. Subsequently, we sought to elucidate the potential mechanisms underlying NagZ’s contribution to the virulence of *E. cloacae*.

In terms of mortality and inflammation, the significant reduction in virulence associated with NagZ knockout represents the primary positive outcome of this study. Previous studies have indicated that the deletion of NagZ does not lead to a significant change in the virulence of *Pseudomonas aeruginosa* towards *Galleria mellonella* [14,37]. However, in a mouse model, the virulence of NagZ knockout *Pseudomonas aeruginosa* is markedly decreased compared to the wild type strain [38]. Our research demonstrates that following NagZ deletion, *E. cloacae* exhibits a substantial decrease in virulence both in vitro (towards phagocytic cells) and in vivo (for *Galleria mellonella* larvae and mice). It is important to note that the mechanisms governing bacterial virulence may vary across different infection models, as previously reported [39].

Peptidoglycan recycling serves multiple crucial physiological functions within bacterial cells, enhancing their ability to survive, adapt, and maintain optimal fitness levels. Although it is not a novel observation that deactivating various targets in the cell wall cycle reduces toxicity in different species [13,40,41], the underlying mechanisms, including the modes of action of lysozyme and peptidoglycan recognition proteins, still require further clarification [13]. Currently, there are some hypotheses regarding these mechanisms. For instance, disruptions in peptidoglycan cycling may impact bacterial energy metabolism, potentially affecting bacterial vitality within the host [14]. Alternatively, it could be linked to the accumulation of specific cell wall fragments that hinder virulence. This theory has been previously proved in *Salmonella spp*. research [35], indicating that the build-up of certain peptidoglycan derivatives may suppress the expression of virulence genes by binding to transcriptional regulatory factors, rather than damaging the recycling process itself [13,35,42]. However, an opposing viewpoint is also conceivable: in cases of impaired peptidoglycan recycling, the production of certain fragments that serve as signals for the activation of virulence genes may decrease, thereby reducing pathogenicity [13]. Our research, on the other hand, found that the knockout of NagZ affects bacterial nucleotide metabolism processes, particularly purine nucleotide metabolism, which is the first report of NagZ involvement in purine nucleotide metabolism.

c-di-GMP, an important purine nucleotide metabolite, plays a role in regulating bacterial traits such as toxicity and invasiveness. The revelation and functional examination of c-di-GMP have ushered in a novel era in the exploration of second messenger signalling mechanisms in prokaryotes, unveiling a level of intricacy akin to that observed in eukaryotes [32,43,44]. c-di-GMP production is primarily facilitated by diguanylate cyclases (DGCs), mediated by their GGDEF domains, while its degradation is carried out by specialized phosphodiesterases (PDEs), which can feature either EAL or HD-GYP domains [45,46]. Typically, these signalling enzymes exhibit a modular domain structure. Various N-terminal domains, often acting as anchors for these enzymes within the cytoplasmic membrane, serve as signal receptors, responding to signals that are frequently not yet fully understood. These DGCs and PDEs engage in a competitive regulation of c-di-GMP levels, which interacts with a wide array of diverse effector components. These components include various proteins that govern diverse targets through direct interactions or riboswitches located in the upstream regions of mRNAs, impacting either transcriptional elongation or translation [47]. These effector-target systems can readily respond to overall shifts in the cellular reservoir of c-di-GMP. Moreover, localized c-di-GMP signalling has been documented, especially in bacterial species possessing multiple DGCs and PDEs. Among them, certain enzymes can exhibit remarkably specific functions due to their direct interactions with effector/target systems [48–50]. The targets regulated by c-di-GMP play crucial roles in fundamental cellular and physiological processes, including the formation of highly antibiotic-resistant biofilms, mobility, virulence, cell cycle progression, and development [34,51].

In this study, we observed a significant increase in c-di-GMP levels in *E. cloacae* following the knockout of NagZ. Previous research has established a positive correlation between c-di-GMP levels and bacterial biofilm formation and adhesion [33]. To further investigate the influence of NagZ on biofilm formation and adhesion, we conducted a series of experiments. First, we assessed biofilm formation using crystal violet staining and found that NagZ-deficient *E. cloacae* strains exhibited a remarkable increase in biofilm formation compared to the wild-type strains. Additionally, we conducted adhesion assays, confirming a notable enhancement in adhesion capabilities in NagZ-deficient strains. To elucidate the molecular mechanisms underlying these observations, transcriptome sequencing was performed. The analysis revealed a significant downregulation in the expression of the unknown functional gene EC_03795 in NagZ-deficient *E. cloacae* strains. This finding suggests that alterations in c-di-GMP levels resulting from NagZ deficiency may be mediated, at least in part, by the regulatory activity of EC_03795.

Notably, EC_03795 features an EAL domain at its C-terminal region, a protein domain commonly found in certain enzymes referred to as PDEs. The EAL domain is responsible for catalysing the hydrolysis of c-di-GMP into linear GMP molecules [52]. Moreover, the N-terminal portion of EC_03795 encompasses a MASE1 domain, which is a putative sensory domain initially characterized by its possession of eight transmembrane segments. This domain is often present at the N-termini of PDEs and histidine sensor kinases and is prevalent in various bacterial groups, including gamma-, beta-, and alpha-proteobacteria, as well as Cyanobacteria [53]. Previous research has elucidated the dual functionality of the MASE1 domain in *Escherichia coli*. It not only serves as an activator of DgcE, one of the diguanylate cyclases responsible for synthesizing c-di-GMP, but also continuously engages in the degradation of DgcE [54]. To determine whether NagZ regulates the virulence of *E. cloacae* through the mediation of EC_03795, we conducted complementation experiments in NagZ-deficient *E. cloacae*. The results demonstrated that the complementation of EC_03795 rescued the elevated c-di-GMP levels and reduced virulence caused by NagZ deficiency. Further investigations revealed that, similar to the NagZ-deficient, the c-di-GMP levels in EC_03795-deficient *E. cloacae* were significantly elevated, which was accompanied by a decrease in virulence. Furthermore, upon complementation of EC_03795, the c-di-GMP levels and virulence in EC_03795-deficient *E. cloacae* were significantly rescued.

In summary, this study represents a significant advancement in our understanding of the regulatory mechanisms in *E. cloacae* virulence. We have successfully identified NagZ’s regulatory role, mediated through the previously unknown functional gene EC_03795 and its interaction with c-di-GMP. This discovery sheds light on potentially widespread mechanisms influenced by dynamic changes in c-di-GMP concentration, providing valuable insights for future research in bacterial virulence regulation. However, our study leaves several intriguing questions unanswered. Firstly, the precise mechanism by which NagZ regulates the expression of EC_03795 and the involvement of any intermediate regulatory factors remain unclear. Secondly, EC_03795 contains an MASE1 domain at its N-terminus and an EAL domain at its C-terminus. However, our research results indicate that EC_03795 negatively regulates c-di-GMP. Does this suggest that the functionality of the MASE1 domain at the N-terminus of EC_03795 has degenerated in *E. cloacae*? Addressing this knowledge gap will be a crucial focus of our future investigations.

## Supplementary Material

Supplemental Material

## Data Availability

The data that support the findings of this study are openly available in the figshare repository at https://doi.org/10.6084/m9.figshare.25898071.v1. The raw data of Transcriptome Sequencing data have been deposited in the GenBank repository under BioProject ID PRJNA1113565 (http://www.ncbi.nlm.nih.gov/bioproject/1113565)
