# Supplementary material for: NagZ modulates the virulence of E. cloacae by acting through the gene of unknown function, ECL_03795
Source: Virulence. 2024 Jun 24;15(1):2367652. doi: 10.1080/21505594.2024.2367652 (PMC11197897; doi:10.1080/21505594.2024.2367652)
Supplement: Supplemental Material [file KVIR_A_2367652_SM7196.zip › Figure S1.docx]

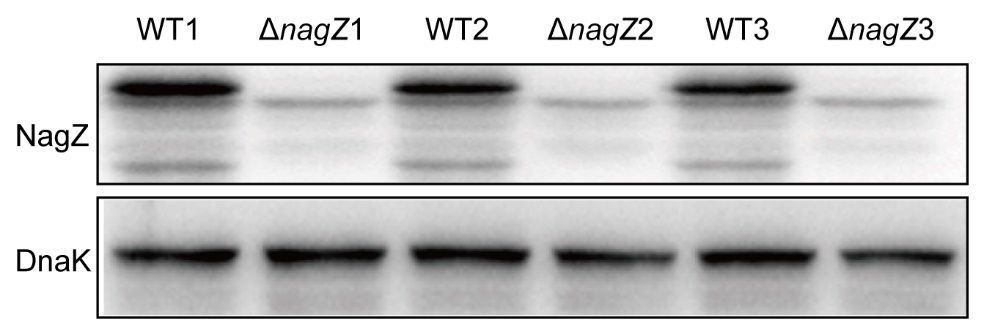


**Fig. S1** Western blot analysis of *nagZ* protein expression in WT strains and Δ*nagZ* strains. DnaK as the internal control. WT: wild type *E. cloacae,* Δ*nagZ: nagZ* knockout *E. cloacae*.
