# Supplementary material for: NagZ modulates the virulence of E. cloacae by acting through the gene of unknown function, ECL_03795
Source: Virulence. 2024 Jun 24;15(1):2367652. doi: 10.1080/21505594.2024.2367652 (PMC11197897; doi:10.1080/21505594.2024.2367652)
Supplement: Supplemental Material [file KVIR_A_2367652_SM7196.zip › Figure S2.docx]

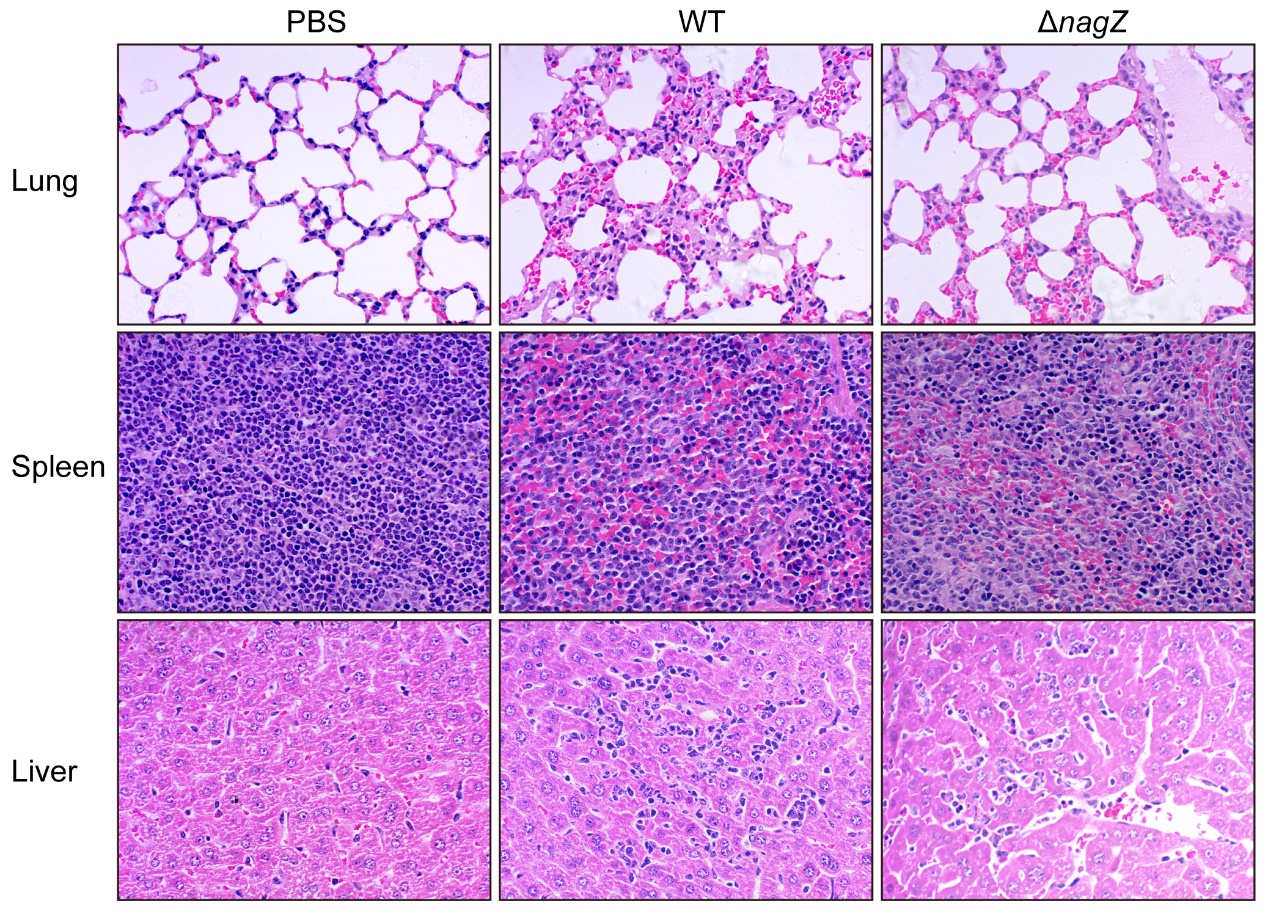


**Fig S2.** The role of NagZ in inducing inflammation by *E. cloacae* in mice was investigated using hematoxylin and eosin staining. Mice treated with PBS did not show significant inflammation in the lungs, spleen, or liver. The WT strain caused substantial lung changes, with noticeable congestion and inflammatory cell infiltration. The spleen displayed marked congestion, and the liver had a significant buildup of inflammatory cells. Conversely, mice infected with the Δ*nagZ* strain had moderate lung changes, including mild congestion and inflammatory cell aggregation. Furthermore, compared to the WT group, Δ*nagZ*-infected mice had reduced inflammation in the spleen and liver. WT: wild type *E. cloacae,* Δ*nagZ: nagZ* knockout *E. cloacae*
